# Estimation of carcass chemical composition in beef-on-dairy cattle using dual-energy X-ray absorptiometry (DXA) scans of cold half-carcass or 11th rib cut

**DOI:** 10.1093/jas/skad380

**Published:** 2023-11-09

**Authors:** Caroline Xavier, Isabelle Morel, Frigga Dohme-Meier, Raphael Siegenthaler, Yannick Le Cozler, Sylvain Lerch

**Affiliations:** Ruminant Nutrition and Emissions, Agroscope, 1725 Posieux, Switzerland; PEGASE INRAE-Institut Agro Rennes-Angers, 16 Le Clos, 35590 Saint-Gilles, France; Ruminant Nutrition and Emissions, Agroscope, 1725 Posieux, Switzerland; Ruminant Nutrition and Emissions, Agroscope, 1725 Posieux, Switzerland; Research Contracts Animals, Agroscope, 1725 Posieux, Switzerland; PEGASE INRAE-Institut Agro Rennes-Angers, 16 Le Clos, 35590 Saint-Gilles, France; Ruminant Nutrition and Emissions, Agroscope, 1725 Posieux, Switzerland

**Keywords:** Carcass quality, crossbreeding, growth, imaging technology, phenotyping, ruminant

## Abstract

The aim of the present study was to estimate the chemical composition (water, lipid, protein, mineral, and energy contents) of carcasses measured postmortem using dual-energy X-ray absorptiometry (**DXA**) scans of cold half-carcass or 11th rib cut. One hundred and twenty beef-on-dairy (dam: Swiss Brown, sire: Angus, Limousin, or Simmental) bulls (*n* = 66), heifers (*n* = 42), and steers (*n* = 12) were included in the study. The reference carcass composition measured after grinding, homogenization, and chemical analyses was estimated from DXA variables using simple or multiple linear regressions with model training on 70% (*n* = 84) and validation on 30% (*n* = 36) of the observations. In the validation step, the estimates of water and protein masses from the half-carcass (*R*^2^ = 0.998 and 0.997; root mean square error of prediction [RMSEP], 1.0 and 0.5 kg, respectively) and 11th rib DXA scans (*R*^2^ = 0.997 and 0.996; RMSEP, 1.5 and 0.5 kg, respectively) were precise. Lipid mass was estimated precisely from the half-carcass DXA scan (*R*^2^ = 0.990; RMSEP = 1.0 kg) with a slightly lower precision from the 11th rib DXA scan (*R*^2^ = 0.968; RMSEP = 1.7 kg). Mineral mass was estimated from half-carcass (*R²* = 0.975 and RMSEP = 0.3 kg) and 11th rib DXA scans (*R*^2^ = 0.947 and RMSEP = 0.4 kg). For the energy content, the *R*^2^ values ranged from 0.989 (11th rib DXA scan) to 0.996 (half-carcass DXA scan), and the RMSEP ranged from 36 (half-carcass) to 55 MJ (11th rib). The proportions of water, lipids, and energy in the carcasses were also precisely estimated (*R*^2^ ≥ 0.882) using either the half-carcass (RMSEP ≤ 1.0%) or 11th rib-cut DXA scans (RMSEP ≤ 1.3%). Precision was lower for the protein and mineral proportions (*R*^2^ ≤ 0.794, RMSEP ≤ 0.5%). The cattle category (sex and breed of sire) effect was observed only in some estimative models for proportions from the 11th rib cut. In conclusion, DXA imaging of either a cold half-carcass or 11th rib cut is a precise method for estimating the chemical composition of carcasses from beef-on-dairy cattle.

## Teaser

Phenotyping the chemical composition (water, lipid, protein, and mineral contents) of carcasses allows for an understanding of bovine growth physiology and could be used in commercial slaughterhouses to determine the commercial values of carcasses. Owing to the present investigation, dual-energy X-ray absorptiometry scans of a cold half-carcass or 11th rib cut were calibrated as accurate and precise methods for estimating carcass chemical composition in a fast, reproducible, and nondestructive way.

## Introduction

Estimations of the tissue and chemical compositions of beef carcasses are used in many carcass-grading systems worldwide (e.g., EUROP in European Union [Regulation (EU) No 1308/2013 of the European Parliament and of the Council, 2013], AUS-MEAT in Australia [Australian government, Department of Agriculture, Fisheries and Forestry, 2019], and CHTAX in Switzerland [Federal Office for Agriculture, 2021]) to determine their commercial values and further valorization in the market. Their high-precision quantification is also of interest from the perspective of genetic selection and in research on animal nutrition and carcass quality. Nonetheless, beef carcass composition is mostly assessed qualitatively by visual or manual grading. Among alternative methods, Scholz et al. (2015) and Silva et al. (2020) reviewed imaging techniques, including ultrasonography, magnetic resonance imaging (**MRI**), or X-ray imaging (computed tomography [**CT**] or dual-energy X-ray absorptiometry [**DXA**]). Ultrasonographic techniques are portative and can be used either in vivo or postmortem to assess the carcass composition, but they only provide a local appraisal of subcutaneous tissue depths. They are also sensitive to the human operator effect during both the image acquisition and treatment steps (Schröder and Staufenbiel, 2006; Scholz et al., 2015). Magnetic resonance imaging and CT technologies provide precise information on the whole or a part of the body from three-dimensional scans, but their use is almost exclusively limited to the research context because of the expensiveness of these devices that require a confined environment (i.e., safety measures regarding magnetic fields and X-ray radiations for MRI and CT, respectively; Scholz et al., 2015; Lerch et al., 2021). As for ultrasonography, MRI and CT are also sensitive to operator expertise in image post-acquisition treatment (Scholz et al., 2015; Silva et al., 2020). Dual-energy X-ray absorptiometry technology uses two different X-ray photon energy levels to provide a scanned image of a whole animal or carcass (Kasper et al., 2021). It provides high precision for the determination of body or carcass composition, similar to CT and MRI, but in comparison, DXA requires no real expertise for image acquisition and treatment, is less time-consuming, and has a much lower X-ray radiation level than CT (Scholz et al., 2015). All these advantages have resulted in large-scale developments, with the current use of DXA in commercial sheep slaughterhouses, even for routine operations, especially in Australia (Gardner et al., 2018; Calnan et al., 2021). In addition, in a research context, DXA has been successfully used to estimate the tissue composition of carcasses from physical dissection in pigs (Suster et al., 2004; Soladoye et al., 2016; Kipper et al., 2019), beef (López-Campos et al., 2018; Ségura et al., 2021), and sheep (Clarke et al., 1999; Mercier et al., 2006). Some interest has also been dedicated to the use of DXA for the estimation of chemical composition (water, lipid, protein, mineral, and energy contents) in pigs (Lukaski et al., 1999; Suster et al., 2003; Soladoye et al., 2016; Kasper et al., 2021), and sheep (Dunshea et al., 2007; Pearce et al., 2009; Hunter et al., 2011).

To the best of our knowledge, in cattle, the determination of carcass chemical composition by DXA has been calibrated only on a small group of cattle of two types (calves and cows; Xavier et al., 2022). Obviously, the large size of beef carcasses limits the feasibility of performing DXA in a slaughterhouse (Calnan et al., 2021). An alternative method may be the estimation of the composition of the whole beef carcass using only a DXA scan of a small portion of it. Indeed, the single or multiple rib-cut dissection method is a common and precise way of estimating the tissue or chemical compositions of the whole carcass, especially for adipose tissue and lipid contents, but its precision is often less satisfactory for the estimation of bone and mineral contents (Hankins and Howe, 1946; Geay et al., 1969; Fiems et al., 2005; Xavier et al., 2022; Lerch et al., 2023). In addition, Mitchell et al. (1997) and Ribeiro et al. (2011) successfully estimated the chemical compositions of 9–11th rib cuts using a DXA scan. Therefore, we hypothesized that the chemical composition of the whole beef carcass can be estimated using a DXA scan of a single-rib cut.

The aim of this study was to predict the chemical composition of cold half-carcasses according to lean, fat, and bone mineral content (**BMC**) masses or proportions measured from DXA scans of cold half-carcasses or 11th rib cuts. Carcasses from 120 growing bulls, heifers, and steers from three beef-on-dairy crossbreeds, achieving more or less intensive growth with different levels of feed intensity and slaughtered at different ages and body weights (**BWs**), were analyzed. Beef-on-dairy cattle are the most commonly used in specialized beef farming systems of several countries such as Switzerland (Lerch et al., 2020b), with a currently growing interest in beef meat production systems elsewhere due to economic and environmental advantages (Faverdin et al., 2022).

## Materials and Methods

All the procedures performed on the animals were approved by the ethics committee of the Fribourg canton of Switzerland (No. 2016_48_FR, 2020_45_FR, and 2020_03_FR).

### Animals and diets

The study was conducted at the experimental farm and slaughterhouse of Agroscope Posieux (Switzerland) from November 7, 2018, to March 10, 2022. A total of 66 bulls, 42 heifers, and 12 steers were used (castrated and dehorned in accordance with Swiss regulations). The cattle were from seven categories (sex × breed of sire): bulls and heifers were from one-third of each, either a crossbreed between Brown Swiss as a dam and Angus, Limousin, or Simmental as the sire (*n* = 22 bulls and 14 heifers of each), whereas steers were only from Brown Swiss as dam and Limousin as the sire (*n* = 12, Supplementary Table S1). All animals were purchased from Swiss commercial dairy farms at 4 to 6 wk of age (65 to 75 kg BW). More details on their diets are presented in Supplementary Information S1.

Fifty-two cattle (12 bulls and 4 heifers of each breed of sire; 4 Brown Swiss × Limousin steers) were slaughtered at 514 ± 18 kg BW and 384 ± 73 days old (mean ± SD), corresponding to a hot carcass weight (**CW**) of 260 to 320 kg. Sixty-eight cattle (10 bulls and 10 heifers of each breed of sire; 8 Brown Swiss × Limousin steers) between 59 and 453 kg BW, corresponding to 219 ± 111 days old, were serially slaughtered. The seven categories of sex × sire breed were used further to classify the individual cattle (Supplementary Table S1).

### Reference postmortem chemical composition measurements

The animals were slaughtered by stunning with captive bolts, followed by exsanguination in accordance with legally defined procedures. The BW was determined before and after exsanguination. Carcasses were dressed after removal of the head, lower legs, tail, hide, and viscera, in accordance with classic commercial procedures. They were then split along the spinal column into two equal parts, individually weighed (hot half-CWs), and further chilled at 4 °C for 24 h before reweighing (cold weights).

The chemical composition of the left half-carcass was determined after thorough grinding and homogenization procedures, as described by Xavier et al. (2022). Two 250-g frozen aliquots of ground carcass homogenate were lyophilized (duplicate DM determination) and finely ground with liquid nitrogen using a knife mill (Grindomix GM200, Retsch, Haan, Germany). Laboratory DM (3 h at 104 °C), mineral (550 °C until constant weight), lipid (petroleum ether extraction), protein (Dumas combustion), and energy contents (adiabatic calorimetry) were further determined in duplicate, in accordance with the method described by Xavier et al. (2022).

### Dual-energy X-ray absorptiometry scan

**Figure 1.**
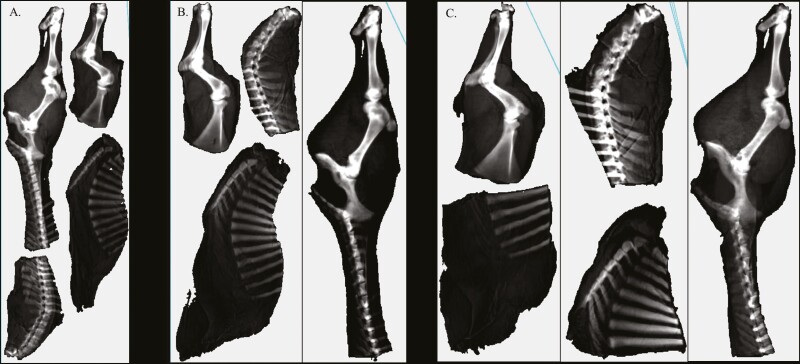
Left half-carcass cut for dual-energy X-ray absorptiometry (DXA) scans: (A) 17 to 46 kg, (B) 52 to 93 kg, and (C) heavier half-carcasses.

The day after the animals were slaughtered, a DXA scanner GE Lunar (iDXA Model, General Electric Medical Systems, Glattbrugg, Switzerland) was used to scan the cold left half-carcass, and the left 11th rib cut, that was sampled as described by Lerch et al. (2023). The DXA scanner used pencil beam technology with X-ray spectra at two different photon energy levels. Before each scan series, it was ensured that the DXA system was in optimal calibration using the quality insurance block phantom provided by General Electric Medical Systems, following the manufacturer’s instructions. The half-carcass scan was performed using the human total body mode (100 kV, 0.188 mA) for 15 min per scan and the 11th rib-cut scan using the small animal body mode (100 kV, 0.188 mA) for 2 to 3 min. Scanning of the whole half-carcass required up to three successive DXA scans after cutting the half-carcass into five cuts because the examination table was not long enough (one scan from 17 to 46 kg, two scans from 52 to 93 kg, and three scans for heavier cold half-carcasses were required; Figure 1). Fat, lean, and BMC mass variables were extracted after image treatment in right-arm mode (Hunter et al., 2011) using the enCORE software (General Electric Medical Systems, Glattbrugg, Switzerland). Artifacts were checked individually and corrected with the right assignment to the corresponding tissue.

### Statistical analyses

Statistical analyses were performed using the R software (version 3.6.3, “Stats” package, R Core Team, 2020). The entire dataset of 120 individuals was split into a training dataset (70% of the entire dataset, *n* = 86) and a validation dataset (30% of the entire dataset, *n* = 34). Both datasets contained all cattle categories (Supplementary Table S1), with equivalent means, SD, and ranges in each variable (Table 1). Simple and multiple linear regressions were performed on the training dataset to estimate the reference carcass chemical composition from the cold half-CW with either the half-carcass or 11th rib-cut DXA scan variables. The variables were selected on the basis of the lowest Bayesian information criterion (BIC function of the “nlme” R package; Pinheiro et al., 2020) among all variables (i.e., cold half-CW and carcass or rib DXA fat, lean, and BMC) in combination, with the effect of the category of cattle (sex × breed of sire, *n *= 7) on the intercept whenever significant (*P* < 0.05). A deviation contrast (“contr.sum”) was applied to take into account the unbalanced group sizes in the linear regression models. Considering the effect of cattle category allowed comparison of both the effect of sex within a specific class of sire’s breed and the effect of the breed of sire within a specific class of sex. For each model selected, the significance (*P* < 0.05) or tendency effects (*P* < 0.10) of the variables were checked with type 3 analysis of variance (R package “car”; Fox and Weisberg, 2019). The estimated marginal means were computed with the R package “emmeans” (Lenth, 2022) in the model with a factor variable. The model parameters presented for the training step are the coefficient of determination (*R²*), the root mean square error (**RMSE**), and the residual coefficient of variation (**rCV**, %; calculated as the ratio of RMSE to the mean of the dependent variable).

**Table 1.** Carcass and 11th rib-cut weights, chemical composition, and dual-energy X-ray absorptiometry (DXA) variables of beef-on-dairy bulls, heifers and steers from Brown Swiss as dam and Angus, Limousin, or Simmental as the sire for the model training and validation datasets

	Model training dataset (*n* = 84)				Model validation dataset (*n* = 36)			
Item	Mean	SD	Min	Max	Mean	SD	Min	Max
Pre-slaughter body weight (BW), kg	372	154	59	531	363	152	74	536
Hot carcass weight (HCW), kg	207	89	34	313	199	87	42	317
Carcass yield (HCW/BW), %	55.3	2.8	48.4	61.2	54.7	3.1	48.2	62.3
Left cold 11th rib weight, g	958.4	433.7	162.1	1,703.0	893.6	398.0	200.8	1,590.0
Left cold half-carcass, kg								
Water	64.4	26.5	12.1	100.9	62.0	25.2	14.5	99.9
Lipids	13.7	9.3	0.8	36.6	13.1	9.2	1.3	33.2
Proteins	19.1	8.1	3.1	30.5	18.3	7.7	4.0	30.6
Minerals	4.3	1.8	0.8	7.1	4.2	1.8	0.8	7.7
Total mass	101.8	44.2	16.8	155.4	97.7	42.8	20.6	157.2
Energy, MJ	988	532	105	1,970	943	526	146	1,882
Cold carcass, %								
Water	64.6	4.0	53.9	71.9	64.8	3.9	57.0	71.2
Lipids	12.0	4.9	4.9	25.9	11.8	4.6	5.7	22.4
Proteins	19.0	0.9	16.4	20.7	18.9	0.9	16.8	21.1
Minerals	4.3	0.5	3.4	5.8	4.4	0.5	3.6	5.4
Energy, MJ/kg	9.1	1.8	6.2	14.0	9.0	1.6	6.7	12.7
Left cold half-carcass DXA scans, kg								
Fat	11.9	8.2	1.0	32.0	11.0	7.8	1.2	27.9
Lean	86.5	36.0	15.4	138.1	83.4	34.3	19.0	135.0
Bone mineral content	4.5	1.9	0.7	7.1	4.4	1.8	0.8	7.3
Total mass	102.9	44.6	17.1	157.3	98.8	43.2	21.0	159.0
Left cold half-carcass DXA scans, %								
Fat	10.3	4.2	4.9	22.5	9.8	3.7	5.2	18.8
Lean	85.3	4.1	73.7	90.7	85.7	3.6	77.2	90.5
Bone mineral content	4.4	0.5	3.4	5.8	4.5	0.6	3.6	5.7
Left cold 11th rib-cut DXA scans, g								
Fat	231.5	143.9	28.7	610.9	214.4	126.4	41.0	461.2
Lean	673.2	291.6	125.4	1,134.7	626.8	264.4	150.5	1,152.9
Bone mineral content	41.7	18.8	6.2	75.0	39.9	17.2	7.0	72.8
Total mass	946.4	425.8	160.8	1,671.1	881.2	389.1	198.9	1,567.3
Left cold 11th rib-cut DXA scans, %								
Fat	23.2	6.8	12.6	45.7	23.0	6.0	12.8	39.5
Lean	72.4	6.6	50.7	83.2	72.4	5.9	56.3	81.7
Bone mineral content	4.4	0.7	2.6	6.3	4.5	0.9	2.8	7.9

At the validation step, model accuracy was assessed on the basis of the mean bias (**MB**), giving an indication if, on average, the model estimation underestimated (MB < 0) or overestimated (MB > 0) the current observation (Tedeschi, 2006). Model precision was assessed on the basis of the *R*², RMSE of prediction (**RMSEP**), and rCV of prediction (**rCVP**). In addition, the mean square error of prediction (**MSEP**) was decomposed into the error of central tendency (**ECT**), error due to regression (**ER**), and error due to disturbances (**ED**) according to Tedeschi (2006). The ECT qualified the overall bias of prediction (if the average predicted value coincided with the average observed value). The ER assessed the error due to the deviation of the regression slope, while the ED represented the unexplained variance that could not be accounted for by the linear regression. When low, the ED was also used as an indicator of lack of fit.

## Results

### Carcass chemical composition and DXA variables

**Figure 2.**
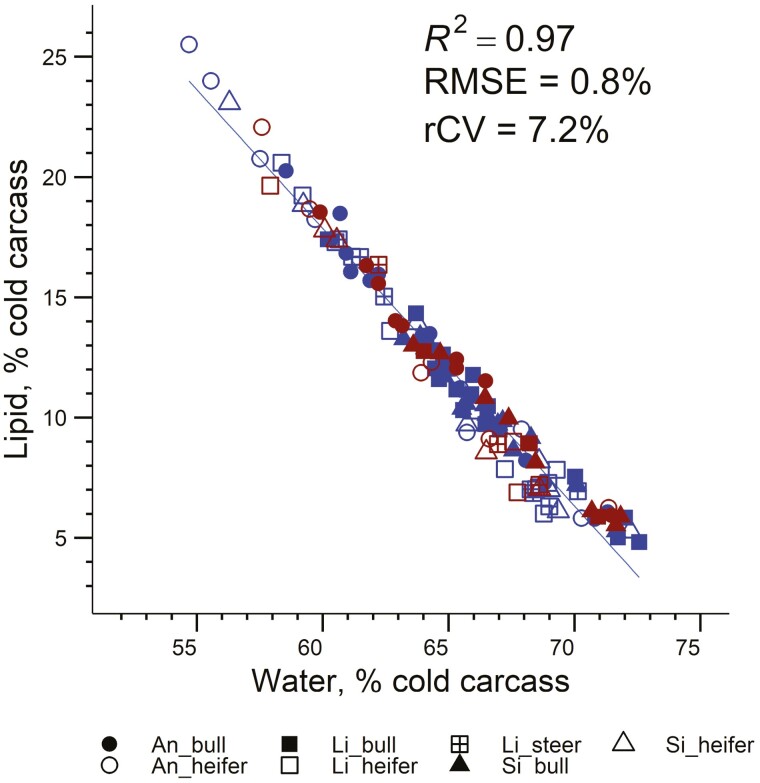
Relationship between water and lipid proportions in the cold half-carcasses of 120 beef-on-dairy bulls, heifers, and steers with Brown Swiss as dam and Angus (An), Limousin (Li), or Simmental (Si) as the sire. Individual cattle are represented in blue for the training dataset (*n* = 86) and in red for the validation dataset (*n* = 34). *R*², coefficient of determination; RMSE, root mean square error; rCV, residual coefficient of variation.

The BW, CW and carcass yield (hot CW-to-BW ratio), and chemical composition are presented in Table 1. By design, the mean, SD, and range in those variables were comparable between the training and validation datasets. When all the observations from the two datasets were combined (*n* = 120), hot CW ranged from 34 to 317 kg, and the carcass yield was 55.1% ± 2.9%. The left cold half-carcass was composed of 63.7 ± 26.1 kg of water, 18.9 ± 8.0 kg of proteins, 13.6 ± 9.2 kg of lipids, 4.3 ± 1.8 kg of minerals, and 975 ± 529 MJ of energy. This corresponded to 64.7% water, 18.9% proteins, 11.9% lipids, 4.3% minerals, and 9.1 MJ energy per kg of fresh matter. The water and lipid proportions in the cold half-carcass were highly negatively correlated (*r* = −0.984, *P* < 0.001, *n* = 120; Figure 2). The values of the DXA variables measured on the left cold half-carcass scans were 11.6 ± 8.0 kg fat mass, 85.6 ± 35.4 kg lean mass, and 4.5 ± 1.8 kg BMC mass (*n* = 120). The coefficients of variation (calculated as the ratio of the SD to the mean) were of similar magnitude for the DXA lean and BMC masses, and the chemical water, protein, and mineral masses (approximately 41%). Similarly, comparable CVs were recorded between DXA fat mass and chemical lipid mass (approximately 68%). The DXA 11th rib scan was composed of 659.3 ± 283.4 g lean, 226.4 ± 138.6 g fat, and 41.2 ± 18.3 g BMC masses (*n* = 120). The mean DXA rib composition was fatter than the DXA half-carcass composition (23.1% vs. 10.2%), resulting in lean proportions of 72.4% and 85.4%, respectively.

### Carcass chemical composition estimation by DXA

#### Chemical masses

**Figure 3.**
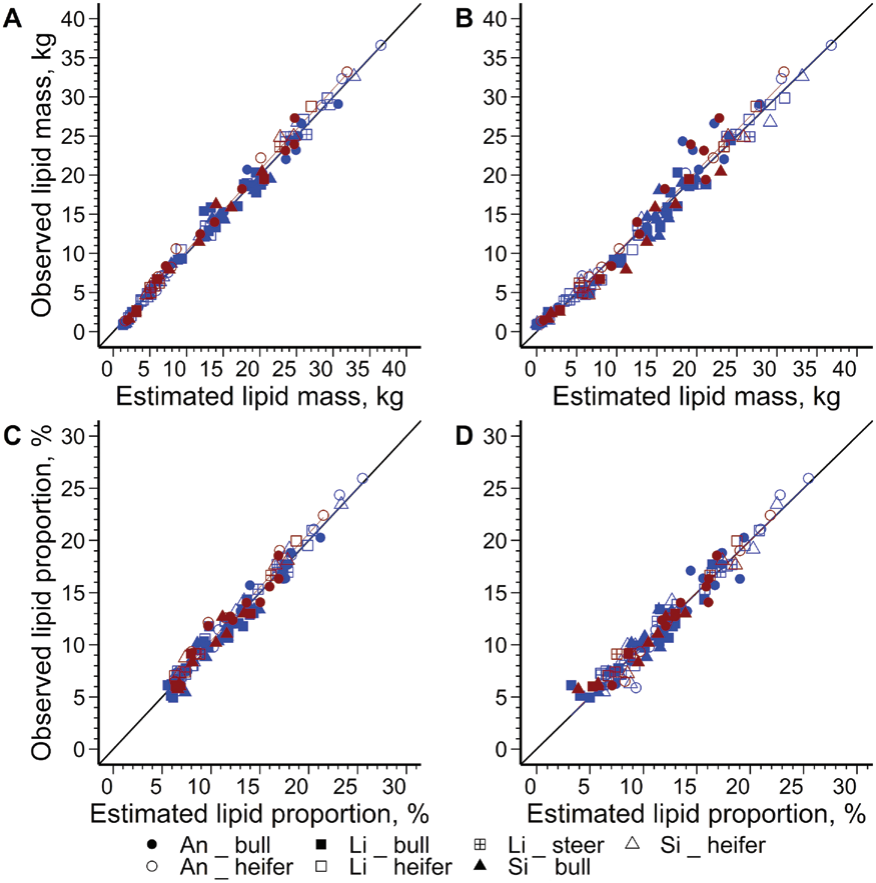
Plots of residuals for the left cold half-carcass lipid mass (A, B) and proportion (C, D) estimations from left cold half-carcass (A, C) or 11th rib-cut (B, D) dual-energy X-ray absorptiometry (DXA) scans in 120 beef-on-dairy bulls, heifers, and steers with Brown Swiss as dam and Angus (An), Limousin (Li), or Simmental (Si) as the sire. Individual cattle and adjustment lines are represented in blue for the training dataset (*n* = 86) and red for the validation dataset (*n* = 34).

The models for estimating the cold half-carcass chemical component masses using the DXA scans of the half-carcasses or 11th rib cuts that were developed on the training dataset are presented in Table 2 and Supplementary Table S2 and the statistics characterizing model precision and accuracy based on the external validation dataset are presented in Table 3 and Supplementary Table S3. In addition, the relationships for estimating lipid mass are illustrated in Figure 3A and B. Overall, the *R*² value was similar, and RMSE only increased by 1.08-fold on average between the model training and validation steps. Furthermore, the precision and accuracy statistics reported in the following text were based on the validation step.

**Table 2.** Most precise estimation equations of cold half-carcass chemical component masses with independent variables from the left cold half-carcass or 11th rib-cut dual-energy X-ray absorptiometry (DXA) scans on the training dataset of 84 beef-on-dairy bulls, heifers, or steers with Brown Swiss as dam and Angus, Limousin, or Simmental as the sire

Chemical component, kg unless stated	Model equation	*R*²	RMSE	rCV, %
From cold half-carcass DXA scan				
Water	*0.140* ^1^ − 0.850 × Fat mass + 0.731 × Left CHC weight	0.999	0.8	1.3
Lipids	0.269 + 1.133 × Fat mass	0.991	0.9	6.5
Proteins	− 0.137 − 0.216 × Fat mass + 0.216 × Left CHC weight	0.997	0.5	2.4
Minerals	*0.104* ^1^ + 0.932 × BMC	0.969	0.3	7.2
Energy, MJ	− *13.94*^1^ + 43.65 × Fat mass + 5.59 × Lean mass	0.997	31	3.1
From cold 11th rib-cut DXA scan				
Water	1.754 + 0.047 × Lean mass − 0.035 × Total mass + 0.631 × Left CHC weight	0.998	1.3	2.0
Lipids	− 2.186 + 0.047 × Fat mass − 0.013 × Lean mass + 0.131 × Left CHC weight	0.975	1.5	10.7
Proteins	*0.085* ^1^ − 0.011 × Fat mass + 0.029 × BMC + 0.200 × Left CHC weight	0.996	0.5	2.6
Minerals	0.354 + 0.032 × BMC − 0.002 × Total mass + 0.045 × Left CHC weight	0.949	0.4	9.2
Energy, MJ	− 86.55 + 1.635 × Fat mass − 0.445 × Lean mass + 9.780 × Left CHC weight	0.990	53	5.4

*R*², coefficient of determination; RMSE, root mean square error; rCV, residual coefficient of variation; CHC, cold half-carcass; BMC, bone mineral content. Fat, lean, BMC, and total masses are acquired from the DXA scans.

^1^When reported in italics, the intercept is not different from 0 (*P* > 0.05).

**Table 3.** Statistics of precision and accuracy for the estimation of cold half-carcass chemical component masses with independent variables from the left cold half-carcass or 11th rib-cut dual-energy X-ray absorptiometry (DXA) scans on the validation dataset of 36 beef-on-dairy bulls, heifers, or steers with Brown Swiss as dam and Angus, Limousin, or Simmental as the sire

Chemical component, kg unless stated	Mean		SD						MSEP decomposition, %		
	Observed	Estimated	Observed	Estimated	MB	*R*²	RMSEP	rCVP, %	ECT	ER	ED
From cold half-carcass DXA scan											
Water	62.0	62.2	25.2	25.2	0.2	0.998	1.0	1.7	2.9	0.2	96.8
Lipids	13.1	12.7	9.2	8.9	− 0.4	0.990	1.0	7.9	14.7	10.9	74.4
Proteins	18.3	18.4	7.7	7.7	0.2	0.997	0.5	2.5	13.8	1.0	85.2
Minerals	4.2	4.2	1.8	1.7	0.0	0.975	0.3	6.7	1.0	10.0	89.0
Energy, MJ	943	933	526	520	− 10	0.996	36	3.8	7.5	2.6	89.9
From cold 11th rib-cut DXA scan											
Water	62.0	62.0	25.2	25.4	0.0	0.997	1.5	2.4	0.1	2.2	97.7
Lipids	13.1	13.0	9.2	8.6	− 0.2	0.968	1.7	12.8	1.2	11.1	87.7
Proteins	18.3	18.5	7.7	7.8	0.2	0.996	0.5	2.9	11.9	4.3	83.7
Minerals	4.2	4.2	1.8	1.7	0.0	0.947	0.4	9.4	0.0	4.0	96.0
Energy, MJ	943	941	526	512	− 2	0.989	55	5.9	0.1	6.7	93.2

SD, standard deviation; MB, mean bias; R^2^, coefficient of determination; RMSEP, root mean square error of prediction; rCVP, residual coefficient of variation of prediction; MSEP, mean square error of prediction; ECT, error in central tendency; ER, error due to regression; ED, error due to disturbances.

The half-carcass DXA total mass highly correlated with the left cold half-CW (*r* > +0.999, *P* < 0.001; slope close from 1 and intercept different from 0; *P* = 0.002), similar to 11th rib weight and DXA total mass (*r* > +0.999, *P* < 0.001; slope close from 1 and intercept not different from 0; *P* = 0.80). The cold left half-CW alone, without the effect of cattle category on the intercept, was used to estimate the chemical composition masses with an *R*^2^ > 0.800, RMSEP ranging from 0.5 kg for minerals to 3.3 kg for water, and rCVP from 5.1% for proteins to 30.5% for lipids. Including the DXA variables from cold half-carcass scans widely improved the adequacy of the estimative equations. Simple linear regressions, without the cattle category effect on the intercept, for the estimation of water and protein masses from DXA lean mass, mineral mass from DXA BMC, and lipid mass and energy content from DXA fat mass had an absolute MB < 3% of the observed mean, *R*^2^ higher than 0.970, RMSEP ranging from 0.3 kg for minerals to 1.0 kg for water and lipids, and rCVP from 1.7% for water to 9.7% for energy (Table 3 and Supplementary Table S3). A slight (*P* > 0.05) improvement in model precision was achieved for lipid and mineral estimations when more than one DXA variable was included (fat mass and BMC, respectively). Conversely, for water and protein masses, multiple linear regressions were selected using the BIC approaches, but the difference between the simple and multiple linear models remained slight (decreases of approximately 0.1 kg in RMSEP and ≤0.2% in rCVP). Only the precision of the energy content estimation was improved relevantly (RMSEP decreased from 92 to 36 MJ and rCV from 9.7% to 3.8% for fat mass simple regression against fat and lean mass multiple linear regression, respectively). The precision decreased when the 11th rib rather than half-carcass DXA variables were used, with an rCVP increase from 1.16-fold for proteins (from 2.5% to 2.9%) to 1.55-fold for energy (from 3.8% to 5.9%, for half-carcass and 11th rib DXA scans, respectively), but with similar *R*² values. By contrast, the accuracy was slightly improved for the 11th rib compared with the half-carcass DXA models, with an absolute MB of 0.6% versus 1.1% of the observed mean. The decomposition of the MSEP confirmed the adequacy of the simple and multiple linear models for either the half-carcass and 11th rib, with < 15% and 11% of the MSEP attributed to ECT and ER, respectively, and the remaining 74% to 98% allocated to ED.

#### Chemical proportions

The models for estimating the carcass chemical component proportions using the DXA scans of the left cold half-carcass or 11th rib that were developed with the training set are presented in Table 4 and Supplementary Table S2, whereas the statistics characterizing model precision and accuracy based on the external validation dataset are presented in Table 5 and Supplementary Table S3. In addition, the relationships for estimating lipid proportion are illustrated in Figure 3C and D. Similarly to the chemical component mass estimations, the *R*² and RMSE were similar between the model training and validation steps (i.e., a mean RMSE increase of 1.03-fold from the training to the validation steps). Therefore, only the statistics from the validation step are reported in the following text.

**Table 4.** Most precise estimation equations of cold carcass chemical component proportions with independent variables from the cold half-carcass or 11th rib-cut dual-energy X-ray absorptiometry (DXA) scans on the training dataset of 84 beef-on-dairy bulls, heifers, or steers with Brown Swiss as dam and Angus (An), Limousin (Li), or Simmental (Si) as the sire

Chemical component, % unless stated	Model equation	*R²*	RMSE	rCV, %
From cold half-carcass DXA scan				
Water	− 9.24 + 0.879 × Lean prop. − 0.011 × Left CHC weight	0.958	0.8	1.3
Lipids	*0.025* ^1^ + 1.076 × Fat prop. + 0.009 × Left CHC weight	0.974	0.8	6.6
Proteins	49.723 − 0.510 × Fat prop. − 0.306 × Lean prop. + 0.006 × Left CHC weight	0.734	0.5	2.4
Minerals	1.624 + 0.679 × BMC prop. − 0.003 × Left CHC weight	0.667	0.3	6.2
Energy, MJ/kg	4.784 + 0.373 × Fat prop. + 0.005 × Left CHC weight	0.969	0.3	3.4
From cold 11th rib-cut DXA scan				
Water	39.37 + 0.403 × Lean prop. − 0.004 × Total mass	0.894	1.3	2.0
Lipids	(− 2.48; − 1.42; − 4.27; − 2.96; − 3.08; − 4.47; − 2.63)^2^ + 0.412 × Fat prop. + 0.006 × Total mass	0.948	1.1	9.2
Proteins	21.45 − 0.107 × Fat prop.	0.657	0.5	2.8
Minerals	3.18 + 0.371 × BMC prop. − 0.005 × Left CHC weight	0.478	0.3	7.7
Energy, MJ/kg	(3.94; 4.17; 3.19; 3.79; 3.58; 3.14; 3.86)^2^ + 0.141 × Fat prop. + 0.022 × Left CHC weight	0.956	0.4	4.2

*R*², coefficient of determination; RMSE, root mean square error; rCV, residual coefficient of variation; CHC, cold half-carcass; BMC, bone mineral content. Fat, lean, and BMC prop. are proportions derived from DXA scans as well as total mass.

^1^When reported in italics, the intercept is not different from 0 (*P* > 0.05).

^2^Intercept in the following order: ×Angus bull; ×Angus heifer; ×Limousin bull; ×Limousin heifer; ×Limousin steer; ×Simmental bull and × Simmental heifer.

**Table 5.** Statistics of precision and accuracy for the estimation of cold half-carcass chemical component proportions with independent variables from the left cold half-carcass or 11th rib-cut dual-energy X-ray absorptiometry (DXA) scans on the validation dataset of 36 beef-on-dairy bulls, heifers, or steers with Brown Swiss as dam and Angus, Limousin, or Simmental as the sire

Chemical component, % unless stated	Mean		SD						MSEP decomposition, %		
	Observed	Estimated	Observed	Estimated	MB	*R²*	RMSEP	rCVP, %	ECT	ER	ED
From cold half-carcass DXA scan											
Water	64.8	65.0	3.9	3.5	0.2	0.932	1.0	1.6	4.2	9.5	86.3
Lipids	11.8	11.4	4.6	4.4	− 0.4	0.959	1.0	8.5	15.8	4.8	79.4
Proteins	18.9	19.1	0.9	0.7	0.2	0.794	0.4	2.3	17.0	19.0	64.1
Minerals	4.4	4.4	0.5	0.4	0.0	0.769	0.2	5.4	0.0	8.8	91.2
Energy, MJ/kg	9.0	8.9	1.6	1.6	− 0.1	0.948	0.4	4.2	7.0	2.1	90.9
From cold 11th rib-cut DXA scan											
Water	64.8	64.9	3.9	3.5	0.0	0.882	1.3	2.0	0.1	8.9	90.9
Lipids	11.8	11.8	4.6	4.5	0.0	0.963	0.9	7.4	0.2	0.3	99.5
Proteins	18.9	19.0	0.9	0.6	0.1	0.659	0.5	2.7	4.3	17.1	78.6
Minerals	4.4	4.4	0.5	0.4	0.0	0.559	0.3	7.5	1.2	13.2	85.5
Energy, MJ/kg	9.0	9.1	1.6	1.7	0.1	0.956	0.4	4.1	5.1	7.0	87.9

SD, standard deviation; MB, mean bias; *R²*, coefficient of determination; RMSEP, root mean square error of prediction; rCVP, residual coefficient of variation of prediction; MSEP, mean square error of prediction; ECT, error of central tendency; ER, error due to regression; ED, error due to disturbances.

The estimation of the chemical component proportions only by left cold half-CW resulted in *R*^2^ values between 0.231 and 0.650, RMSEP of 0.4% to 3.1%, and rCVP of 3.8% to 25.8%. The rCVP was 1.3- to 2.6-fold lower when simple linear regressions involving only one predictive DXA variable (DXA lean for water and proteins, DXA fat for lipids and energy, and DXA BMC for minerals) were performed, ranging from 1.6% for water to 8.6% for lipids (Supplementary Table S3). Those were not relevantly improved further in accuracy or precision after adjustment for multiple linear regression models, with equal absolute MB, and rCVP equal for water or slightly improved for lipids and proteins, decreasing from 0.1 point to 0.7 point, respectively. In the multiple linear regression models, the left cold half-CW was always entered as an additional variable. For protein proportion estimation, three variables were computed in the model: lean and fat proportions, and left cold half-CW. A slight decrease in precision was observed when the half-carcass DXA variables were substituted with those for the 11th rib cut for water, protein, and mineral proportions: the rCVP increased from 1.6% to 2.0% for water, from 2.3% to 2.7% for proteins, and from 5.4% to 7.5% for minerals in half-carcass and 11th rib DXA scan regressions. Conversely, accuracy was slightly improved for the 11th rib compared with half-carcass DXA models for the water, lipid, and protein proportions, with a mean absolute MB of 0.2% and 1.6% of the observed mean. The decomposition of the MSEP confirmed the adequacy of the simple and multiple linear models for proportion estimation, from either the half-carcass and 11th rib, with < 17% and 19% of the MSEP attributed to ECT and ER, respectively, and the remaining 64% to 99% attributed to ED.

### Effect of the cattle category on the estimative model precision

When the category of cattle (sex × sire breed) effect was included in the estimation models for the lipid and energy proportions from the 11th rib DXA variables, the rCVP was decreased by more than 1 point, conversely to a non-relevant improvement, even if significant (*P* < 0.05), for other proportion estimative models from the 11th rib DXA scans. These models are presented in Table 4 and their validations are presented in Table 5. In addition, no relevant improvement of model precision or accuracy due to cattle category inclusion was observed for the estimations of the chemical component masses from the DXA scans of the half-carcass or 11th rib, similar to the component proportions from the half-carcass DXA scans.

## Discussion

### Carcass chemical composition

The cold half-carcass fat-free composition (CW − carcass lipid mass) was almost constant, regardless of the cattle age category, with values of 72% to 76% for water, 19% to 23% for proteins, and 4% to 6% for mineral content. This composition is close to those reported by Fiems et al. (2005); Yan et al. (2009); Lerch et al. (2015, 2021), and Xavier et al. (2022) for fat-free empty body composition. Cold-carcass lipid proportions still varied widely in the present study owing to the inclusion of both a range of cattle age and CW (25 to 557 d old and 34 to 317 kg hot CW), and sexes and breeds of sire of divergent precocities (late-, mid-, and very early-maturing breeds, i.e., Limousin, Simmental, and Angus, respectively). This large-scale BW, age, and carcass fatness are representative of the diversity of beef carcasses in the domestic markets of several countries (Greenwood, 2021).

### Estimation of carcass chemical composition from DXA scans of half-carcass

The *R*^2^ values for the chemical component mass estimations from the half-carcass DXA scans at the validation step were often close to or higher than 0.95 in the present study, in close agreement with the *R*² values reported by others for DXA calibration for beef carcasses, regardless of the reference composition measurement: chemical analyses (Xavier et al., 2022), primary cut tissue dissection (López-Campos et al., 2018), or CT scan (Calnan et al., 2021). The precision obtained was also of a similar range to that for sheep chemical composition (Clarke et *al*., 1999; Dunshea et al., 2007; Hunter et al., 2011). The high *R*^2^ value across the studies could be explained by the large weight range of the carcasses scanned by DXA. López-Campos et al. (2018) and Xavier et al. (2022) studied animals with higher BW ranging from 291 to 663 kg and from 302 to 754 kg, respectively. The range of BW was also important in studies based on other types of animals: 6 to 20 kg for Clarke et al. (1999) and 8 to 28 kg for Dunshea et al. (2007) for growing lambs and adult ewe carcasses, respectively.

The increase in precision with multiple linear models was small compared with that of simple linear regressions using only one DXA variable, which appears to be also suitable. In sheep, Clarke et al. (1999) and Pearce et al. (2009) published simple linear regressions between DXA BMC mass and mineral mass, DXA lean mass and protein mass, or DXA fat mass and lipid mass, with an equivalent *R*^2^ value compared with those obtained by simple regressions in the present study, with the exception of the mineral mass estimation by Pearce et al. (2009), with a lower *R*^2^ value (0.39) in the latter case. These authors also summed the water and protein masses to be closer to the definition of the lean mass provided by DXA, without improving the relationship, than the results of the present study, splitting the estimations of water and protein masses.

The models for the estimation of lipid and mineral masses were similar in accuracy (based on MB) but less precise (based on rCVP) than those for the other chemical components. The estimations of lipid and mineral masses from DXA appeared to be slightly more difficult to determine across species with similar rCV values at the model training step in a former study on sheep (Dunshea et al., 2007) than in the present study. Pearce et al. (2009) also had difficulty estimating mineral mass from sheep carcass DXA scans with an *R*^2^ of 0.39 and an rCV of 27%. Similarly, López-Campos et al. (2018) obtained a lower precision from the DXA BMC mass to predict the bone mass of steer carcasses when calibrated against CT measurement as a reference. The same was noticed by Ségura et al. (2021) in culled cows and Mercier et al. (2006) in lambs for predicting dissected bone mass from the DXA BMC mass, compared with higher precision for the adipose tissue and muscle mass estimations from DXA fat and lean masses, respectively.

Overall, similar absolute MB and rCVP but lower *R*² of the predictive equations were obtained for the proportion compared with mass. This is mostly due to the quasi-constant carcass proportion in some chemical components (especially proteins and minerals), even when a wide range of CW is included, which accordingly decreases the variability of the variable to be predicted. The prediction of lipid proportion by the half-carcass DXA scan was the component with the highest rCVP among all the proportion estimations. This was slightly higher than the values reported by Dunshea et al. (2007) in sheep half-carcass (5.4% and 5.7% for multiple and simple linear regressions, respectively) that were fatter than the beef half-carcass studied presently (30% vs. 12% lipids). Indeed, the RMSE is conversely slightly higher in the study of Dunshea et al. (2007) than in the present study. In addition, using a CT scan as a reference, Calnan et al. (2021) found a higher rCV when calibrating DXA in beef carcass for adipose tissue and muscle proportion estimations than the rCV for lipid and protein proportions in the present study, but it was similar for the bone than for the mineral proportions.

The half-carcasses of goats (containing 5.5 to 8.2 kg of water and 1.7 to 2.5 kg of proteins) were formerly scanned at the Agroscope Posieux experimental facilities (Lerch et al., 2020a), using the same DXA device and following a similar protocol as for beef in the present study. Kasper et al. (2021) also used the same DXA device and protocol to scan pig carcasses (with skin, feet, and head; from 8.9 to 50.8 kg of water and 2.1 to 15.6 kg of proteins). The different estimative DXA models from simple regressions (DXA lean mass for water and protein, DXA fat mass for lipid and energy, and DXA BMC mass for mineral estimates) from these three studies had an *R*^2^ value higher than 0.94 and reflected a high precision of the simple regressions studied, with the exception of the mineral mass estimations in goats (*R*^2^ of 0.69; Lerch et al., 2020a). For protein estimations, the rCV was 2.6% (2.7% for the rCVP on the validation dataset) in the present study, 3.1% in Lerch et al. (2020a), and 4.5% in Kasper et al. (2021). The best rCV for water mass estimation was obtained by Kasper et al. (2021) and in the present study, with 1.5% (1.7% for the rCVP; 3.0% in Lerch et al., 2020a). For lipids and minerals, rCV was more important for Lerch et al. (2020a) than in the present study, with +5.9 and +1.7 points for the lipid and mineral estimations, respectively (no equivalent relationship for Kasper et al., 2021). The slope coefficients of the DXA scan simple regression for chemical component mass estimations for the beef half-carcass (present study) were close to those for the goat half-carcass (Lerch et al., 2020a), with a difference ranging from 4% for proteins to 15% for water, and for pig half-carcass (Kasper et al., 2021), with a difference ranging from 0% for proteins to 3% for water. Therefore, despite the differences in carcass dressing and composition between species, when established using the same DXA device, the equations calibrated for beef cattle in the present study are close to those obtained in pigs by Kasper et al. (2021) and in goats by Lerch et al. (2020a). Such indirect comparison highlights that the same DXA device could have a linear response to a wide range of CW and chemical composition regardless of the species and concomitant way to dress the carcass. This would be a key advantage in the perspective of calibrating and further application of a generic set of estimative models suitable for a large number of species without dealing with species, sex, or breed.

### Estimation of carcass chemical composition from DXA scans of the 11th rib

The precision of the estimative equations from the DXA scan was slightly lower for the 11th rib cut than for the cold half-carcass. For lipid mass and energy content estimations, the rCV from the 11th rib-cut DXA scan was higher in the present study than in the study of Xavier et al. (2022), which may be due to carcasses of mature cattle being richer in lipid in the latter study than in the present study (17% vs. 12%). Including the effect of the category of cattle (sex × breed of sire) in predictive equations from the 11th rib DXA scan improved their precision for lipid and energy proportions, with a decrease in rCVP ranging between 0.2 and 4.5 points.

In upcoming developments, calibrating estimative equations for each breed or crossbreed will not be necessarily needed, but it will be most likely for groups of breeds, as proposed by Miller et al. (2019). Therefore, the use of a single-carcass cut (11th rib here) DXA scan is promising for estimating full carcass composition, as previously demonstrated from dissection or chemical analyses of rib cuts (Hankins and Howe, 1946; Geay et al., 1969; Robelin and Geay, 1976; Fiems et al., 2005). Moreover, DXA scans instead of dissection or chemical analyses alleviated the drawbacks of the latter techniques that are destructive, time-consuming, and sensitive to operator effect on dissection (Lerch et al., 2023).

### Implications for high-throughput monitoring of carcass composition

The DXA scan of a complete carcass would be easier to deal with than the DXA scan of the 11th rib from the perspective of implementation in slaughterhouses at chain speed (Gardner et al., 2018; Connaughton et al., 2020, 2021). However, several technical challenges must be overcome before using on-field DXA for beef carcasses. This requires a safe area around the device to limit the exposure of operators to X-rays, whereas the device should be adapted to beef carcass size (Calnan et al., 2021). Another option would rely on the use of DXA to scan a single-carcass primary cut, but the exact anatomical delimitation of primary cuts depends, among others, on the country, CW, and carcass classification and market segmentation. The rib cut can be a suitable option for collecting fine estimates of carcass chemical composition from several slaughterhouses with only a carcass sample of approximately 1 kg by scanning with a single DXA device potentially located far away from the origin slaughterhouses. In addition, compared with the traditional methods (i.e., rib-cut dissection), the use of DXA limits or suppresses the operator effect. However, this method still requires a cut during the slaughterhouse process, which would have an economic impact on the commercial value of the carcass. Traceability and adequate attribution of the rib to the corresponding carcass would also remain challenging. This on-field application is an opportunity to conduct measurements on a large number of animals and/or to have an overview of the current carcass composition in slaughterhouses at high throughput owing to fast rib-cut DXA scans. The use of a single-rib cut (e.g., the 11th rib) DXA scan can be a suitable option to save time in scanning and to reduce the safe area needed around the device. This could lead to wide implications such as in the field of genetic selection for improving carcass quality. Other imaging methods were recently developed using smartphone photo analyses from rib sections (Santos et al., 2013; Meunier et al., 2021) to estimate the tissue and chemical compositions of this single section of the carcass. These rapidly developing imaging techniques need further insight to estimate the composition of the entire carcass with appropriate calibration, as was done in the present study for DXA imaging. Ultimately, a smartphone-based method would simplify the acquisition of data at a low cost, with no dependence on expensive and immobile devices such as DXA.

## Conclusion

The present study confirms that the DXA imaging technology offers a rapid, objective, accurate, and precise estimation of chemical component masses and proportions in the carcasses of beef-on-dairy cattle. Such a technique is not destructive of the carcass and not dependent on the operator effect. The DXA scan of cold half-carcass was the most precise option for estimating its chemical composition, but the single-11th rib-cut scan remained an accurate and precise alternative and faster option. The estimation models developed in the present study can be used for most types of cattle, within a wide range of BW and carcass composition. However, the effect of cattle category on regression intercepts, especially for the rib-cut DXA scan for chemical proportion estimations, must be studied in detail in the future, and more crossbreed types must be included to improve and expand the developed models.

## Supplementary Material

skad380_suppl_Supplementary_Material

## Data Availability

The individual data of carcass chemical composition in exp. 1 and 2 are available in the Data INRAE repository at https://doi.org/10.57745/EK4FFP. Other data can be accessed by the authors upon reasonable request.

## Abbreviations

AUS-MEAT: Australian carcass classification scheme
BIC: Bayesian information criterion
BMC: bone mineral content
BW: body weight
CHTAX: Swiss carcass classification scheme
CT: computer tomography
CW: carcass weight
DM: dry matter
DXA: dual-energy X-ray absorptiometry
ECT: error of central tendency
ED: error due to disturbances
ER: error due to regression
EUROP: European Union carcass classification scheme
MB: mean bias
MRI: magnetic resonance imaging
MSEP: mean square error of prediction
rCV: residual coefficient of variation
rCVP: residual coefficient of variation of prediction
RMSE: root mean square error
RMSEP: root mean square error of prediction
