# Supplementary material for: Estimation of carcass chemical composition in beef-on-dairy cattle using dual-energy X-ray absorptiometry (DXA) scans of cold half-carcass or 11th rib cut
Source: J Anim Sci. 2023 Nov 9;101:skad380. doi: 10.1093/jas/skad380 (PMC10718802; doi:10.1093/jas/skad380)
Supplement: skad380_suppl_Supplementary_Material [file skad380_suppl_supplementary_material.docx]

**Estimation of carcass chemical composition in beef-on-dairy cattle using dual-energy X-ray absorptiometry (DXA) of cold half-carcass or 11^th^ rib cut**

Caroline Xavier*^,†^, Isabelle Morel*, Frigga Dohme-Meier*, Raphael Siegenthaler^‡^, Yannick Le Cozler^†^, Sylvain Lerch*^,1^

^*^Ruminant Nutrition and Emissions, Agroscope, 1725 Posieux, Switzerland

^†^PEGASE INRAE-Institut Agro Rennes-Angers, 16 Le Clos, 35590 Saint-Gilles, France

^‡^Research Contracts Animals, Agroscope, 1725 Posieux, Switzerland

^1^ Corresponding Author: Sylvain Lerch, sylvain.lerch@agroscope.admin.ch, 0041 58 461 41 29, CH-1725 Posieux

**Supporting Information 1**

**Supplementary Tables 1-3**

**Pages S1-S5**

**Supporting Information 1:** Details of the diets during the breeding and the experiment.

Calves were fed with milk replacer until weaning at 13 weeks of age, completed with concentrate, hay, and corn silage (1^st^ and 2^nd^ experiment) or grass silage (3^rd^ experiment). From 19 ± 3 weeks of age (166 ± 15 kg BW), cattle were fed different diets until slaughter:

- Eighteen (18) bulls received one of two iso-energetic [7.6 MJ net energy for meat production (NEV) per kg of dry matter (DM); Agroscope 2021] and iso-proteic [146 and 148 g crude protein (CP) per kg of DM] total mixed rations (TMR) either composed of (DM basis) 63% corn silage, 7% alfalfa hay (diet A) or 49% corn silage, 21% fermented beet pulp, and 10% grass silage (diet B), all completed with energy and protein concentrates (27% and 18% for rations A and B, respectively) as well as minerals and vitamins (3% and 2%),
- Forty-eight (48) bulls received one of two iso-energetic (7.27 and 7.1 MJ NEV per kg of DM) and iso-proteic (160 and 156 g CP per kg of DM) TMR composed of 38% corn silage with 36% grass silage in diet C, or with 34% grass-alfalfa silage and 1% straw in diet D, completed with peas (11%) and barley (9%) in diet C or faba beans (6%) and triticale (13%) in diet D, as well as corn gluten meal (2%), soybean meal (2% and 3% in diets C and D respectively), and minerals and vitamins (3%) in both C and D diets,
- Fifty-four (54) heifers and steers received successively along growth the same two successive TMR diets E and F (5.9 to 6.1 MJ NEV and 120 to 180 CP per kg of DM) composed of 56–70% grass silage, 19–24% hay, 8–14% corn silage, completed with corn gluten meal and rapeseed meal (2–4% in E only), barley (3–6%), and minerals and vitamins (1%). Among these heifers and steers, 24 from 230–300 kg BW were at pasture on permanent grassland for 16 weeks.

**Reference**

Agroscope. 2021. Feeding recommendations and nutrient tables for ruminants [in French]. In: I. Morel, J.-L. Oberson, P. Schlegel, A. Chassot, E. Lehmann, and J. Kessler. Apports alimentaires recommandés pour le bovin à l’engrais. Dans: Apports alimentaires recommandés pour les ruminants (Livre vert). Octobre Ed. Agroscope, Posieux. 2017, p. 1-27. Available from https://www.agroscope.admin.ch/agroscope/fr/home/services/soutien/aliments-pour-animaux/apports-alimentaires-recommandes-ruminants.html.

| **Supplementary Table 1**: Number of cattle allocated in the different body weight classes at slaughter depending on animal categories (sex × sire breed)^1^, for the model training and validation datasets | | | | | | | | |  |
| --- | --- | --- | --- | --- | --- | --- | --- | --- | --- |
|  |  | Mean (SD) of body weight at slaughter, kg | | | | | |  | |
|  |  | 74 (8) | 164 (6) | 253 (25) | 324 (34) | 405 (38) | 513 (18) | Total | |
| Training dataset | | | |  |  |  |  |  | |
|  | ×Angus bull | 1 | 2 | 1 | 1 | 1 | 7 | 13 | |
|  | ×Angus heifer | 1 |  | 2 | 1 | 1 | 3 | 8 | |
|  | ×Limousin bull | 2 | 1 | 1 | 2 | 2 | 11 | 19 | |
|  | ×Limousin heifer | 2 |  | 3 | 1 | 2 | 3 | 11 | |
|  | ×Limousin steer |  |  | 2 | 2 | 2 | 3 | 9 | |
|  | ×Simmental bull | 1 |  | 2 | 1 | 1 | 9 | 14 | |
|  | ×Simmental heifer | 2 |  | 2 | 2 | 2 | 2 | 10 | |
|  | Total | 9 | 3 | 13 | 10 | 11 | 38 | 84 | |
| Validation dataset | | |  |  |  |  |  |  | |
|  | ×Angus bull | 1 |  | 1 | 1 | 1 | 5 | 9 | |
|  | ×Angus heifer | 1 |  | 2 | 1 | 1 | 1 | 6 | |
|  | ×Limousin bull |  | 1 | 1 |  |  | 1 | 3 | |
|  | ×Limousin heifer |  |  | 1 | 1 |  | 1 | 3 | |
|  | ×Limousin steer |  |  | 2 |  |  | 1 | 3 | |
|  | ×Simmental bull | 1 | 2 |  | 1 | 1 | 3 | 8 | |
|  | ×Simmental heifer |  |  | 2 |  |  | 2 | 4 | |
|  | Total | 3 | 3 | 9 | 4 | 3 | 14 | 36 | |
| ^1^Every cattle were from Brown Swiss as dam. | | | | | | | | |  |

**Supplementary Table 2:** Simple regressions for the estimation of cold half-carcass chemical composition with independent variables from the cold half-carcass dual-energy x-ray absorptiometry (DXA) scan on the training set of 84 beef-on-dairy bulls, heifers or steers with Brown Swiss as dam and Angus, Limousin or Simmental as sire

| Chemical component | | Model equation | *R²* | RMSE | rCV (%) |
| --- | --- | --- | --- | --- | --- |
| Masses, kg | |  |  |  |  |
|  | Water | 0.66 + 0.74 × Lean mass | 0.999 | 0.9 | 1.5 |
|  | Proteins | -0.30 + 0.23 × Lean mass | 0.996 | 0.5 | 2.6 |
|  | Energy, MJ | 234.95 + 63.39 × Fat mass | 0.946 | 123 | 12.6 |
| Proportions, % | |  |  |  |  |
|  | Water | -17.23 + 0.96 × Lean prop. | 0.951 | 0.9 | 1.4 |
|  | Lipids | *0.21*^1^ + 1.14 × Fat prop. | 0.970 | 0.8 | 7.0 |
|  | Proteins | 4.10 + 0.17 × Lean prop. | 0.627 | 0.5 | 2.9 |
|  | Minerals | 1.09 + 0.73 × BMC prop. | 0.590 | 0.3 | 6.8 |
|  | Energy, MJ / kg | 4.92 + 0.41 × Fat prop. | 0.960 | 0.3 | 3.8 |
| Abbreviations: coefficient of determination (*R²*), root mean square error (RMSE), residual coefficient of variation (rCV). Fat, lean and BMC prop. are proportions derived from DXA scan.  ^1^When reported in italics, the intercept is not different from 0 (*P* > 0.05). | | | | | |

**Supplementary Table 3:** Statistics of precision and accuracy for the estimation of cold half-carcass chemical composition from the simple regressions with independent variables from the left cold half-carcass dual-energy x-ray absorptiometry (DXA) scan on the validation dataset of 36 beef-on-dairy bulls, heifers or steers with Brown Swiss as dam and Angus, Limousin or Simmental as sire

|  |  | Mean | |  | SD | |  |  |  |  | MSEP decomposition, % | | |
| --- | --- | --- | --- | --- | --- | --- | --- | --- | --- | --- | --- | --- | --- |
| Chemical component | | Observed | Predicted |  | Observed | Predicted | MB | *R²* | RMSEP | rCVP, % | ECT | ER | ED |
| Masses, kg | |  |  |  |  |  |  |  |  |  |  |  |  |
|  | Water | 62.0 | 62.1 |  | 25.2 | 25.3 | -0.1 | 0.998 | 1.0 | 1.7 | 1.6 | 1.2 | 97.3 |
|  | Proteins | 18.3 | 18.4 |  | 7.7 | 7.7 | -0.2 | 0.996 | 0.5 | 2.7 | 11.5 | 0.5 | 88.0 |
|  | Energy, MJ | 943 | 933 |  | 526 | 495 | 10 | 0.971 | 92 | 9.7 | 1.1 | 10.8 | 88.1 |
| Proportions, % | |  |  |  |  |  |  |  |  |  |  |  |  |
|  | Water | 64.8 | 65.0 |  | 3.9 | 3.4 | -0.2 | 0.931 | 1.1 | 1.6 | 3.7 | 16.0 | 80.3 |
|  | Lipids | 11.8 | 11.4 |  | 4.6 | 4.3 | 0.4 | 0.959 | 1.0 | 8.6 | 15.1 | 8.7 | 76.2 |
|  | Proteins | 18.9 | 19.1 |  | 0.9 | 0.6 | -0.2 | 0.594 | 0.6 | 3.0 | 8.8 | 15.9 | 75.2 |
|  | Minerals | 4.4 | 4.4 |  | 0.5 | 0.4 | 0.0 | 0.792 | 0.2 | 5.2 | 0.0 | 12.9 | 87.0 |
|  | Energy, MJ | 9.0 | 9.0 |  | 1.6 | 1.5 | 0.1 | 0.943 | 0.4 | 4.4 | 6.0 | 6.6 | 87.5 |
| Abbreviations: standard deviation (SD), mean bias (MB), coefficient of determination (*R²*), root mean square error of prediction (RMSEP), residual coefficient of variation of prediction (rCVP), mean square error of prediction (MSEP), error of central tendency (ECT), error due to regression (ER), error due to disturbances (ED). | | | | | | | | | | | | | |
|  |  |  |  |  |  |  |  |  |  |  |  |  |  |
